# *Aspergillus flavus* with Mycovirus as an Etiologic Factor for Acute Leukemias in Susceptible Individuals: Evidence and Discussion

**DOI:** 10.3390/biomedicines13020488

**Published:** 2025-02-17

**Authors:** Cameron K. Tebbi, Eva Sahakian, Bijal Shah, Jiyu Yan, Melanie Mediavilla-Varela, Saumil Patel

**Affiliations:** 1 Children’s Cancer Research Group Laboratory, Tampa, FL 33613, USA; jiyuyanshine@gmail.com; 2 Moffitt Cancer Center, Tampa, FL 33612, USA; eva.sahakian@moffitt.org (E.S.); bijal.shah@moffitt.org (B.S.); melanie.mediavilla-varela@moffitt.org (M.M.-V.); 3 Tampa General Hospital, Tampa, FL 33606, USA; spatel@tgh.org

**Keywords:** leukemia, etiology, leukemogenesis, *Aspergillus flavus*, mycovirus, cancer, carcinogenesis, asthma, agriculture, epidemiology

## Abstract

Several etiologic factors for the development of acute leukemias have been suggested; however, none is applicable to all cases. We isolated a certain mycovirus-containing *Aspergillus flavus* (MCAF) from the home of a patient with acute lymphoblastic leukemia. Repeated electron microscopic evaluations proved the existence of mycovirus in this organism. According to chemical analysis, this organism does not produce any aflatoxin, possibly due to its infestation with mycoviruses. We reported that using the ELISA technique, forty pediatric patients with acute lymphoblastic leukemia (ALL) uniformly had antibodies to the products of MCAF. In contrast, three separate groups of controls, consisting of normal blood donors, individuals with solid tumors, and patients with sickle cell disease, were negative. In vitro exposure of mononuclear blood cells from patients with ALL, in full remission, to the products of MCAF induced redevelopment of cell surface phenotypes and genetic markers characteristic of ALL. The controls were negative. The incubation of normal and ALL cell lines with the products of MCAF resulted in significant cellular apoptosis, changes in the cell cycle, and the downregulation of transcription factors, including PAX-5 and Ikaros (75 and 55 kDa). Fungi are widespread in nature, and many contain mycoviruses. Normally, an individual inhales 1 to 10 fungal spores per minute, while farmers can inhale up to 75,000 spores per minute. It is known that farmers and foresters, who are more exposed to fungi, have a higher rate of acute leukemia. In contrast, asthmatics, most of whom are allergic to fungal agents, and individuals working in office settings have a lower rate. One of the theories for the development of acute leukemia suggests a genetic predisposition followed by exposure to an infectious agent. With the above findings, we propose that mycovirus-containing *Aspergillus flavus* may have an etiological role in leukemogenesis in immune-depressed and genetically susceptible individuals.

## 1. Introduction

Worldwide, leukemias are malignant disorders, ranking as the thirteenth most frequently diagnosed cancer and the tenth cause of death. Based on the GLOBOCAN Cancer data for 2020, globally, leukemia cases constituted 2.5% of all newly diagnosed cancers and 3.1% of cancer deaths [1]. In 2022, over 487,000 new cases of leukemia were diagnosed, and there were 305,000 deaths due to these disorders [2]. According to the National Cancer Institute Surveillance, Epidemiology, and End Results (SEER) database, in 2024, there were an estimated 62,770 cases of leukemia in the United States (US). The age-standardized rate for leukemia is approximately 11 per 100,000. According to the Surveillance, Epidemiology, and End Results (SEER) database, the estimated deaths from leukemia in the US in 2021 were reported to be 23,660, which constitutes 3.9% of all deaths due to cancer. Some statistics reveal that since 2006, the incidence of leukemias has increased; however, the mortality rate due to these disorders has decreased [3]. Worldwide, there is a significant geographical, environmental, and ethnic variation in the rate of leukemias [2,3,4,5,6,7,8,9,10]. These disorders are seen in both sexes and all age groups but are more prevalent in males. Based on the 2018 global statistics, the age-standardized incidence rates for males and females were 6.1 and 4.3 per 100,000, respectively [11]. Statistically, mortality due to leukemia is also higher in males, at 4.3 per 100,000, compared with females, at 2.8 per 100,000 population [11,12].

The distribution of leukemias varies based on a variety of factors, including the type of disease and age. ALL and acute myelogenous leukemia (AML) occur in children and adults, while chronic myelogenous (CML) and chronic lymphocytic (CLL) forms of the disease are generally seen in the older age groups. In the United States, in children, adolescents, and young adults less than 20 years old, the age-adjusted incidence rate of leukemia between 2012 to 2016 was reported to be 4.6 per 100,000. This constitutes 20–30% of all cancers in this age group. Leukemia is the most common childhood cancer, accounting for approximately 30% of cancers diagnosed in those under 15 years of age [1,13]. Acute lymphoblastic leukemia is the most common form in children, accounting for 75% of all leukemias. In contrast, acute myelogenous leukemia, with an incidence of 3–5 cases per 100,000 in the general population, is most prevalent in adults. 

## 2. Pathophysiology

The events leading to the genesis of leukemia are not entirely clear. Physiologically, hematopoietic stem cells (HSCs) are primitive cells capable of self-renewal and differentiation into any of the hemopoietic cell lineages. Acute leukemias are caused by a series of mutations during the complex process of hemopoiesis. Under normal conditions, hemopoiesis begins with hemopoietic stem cells. These can self-renew and differentiate into any of the blood cell lineages. Hemopoietic stem cells must be precisely regulated to maintain normal hemopoiesis. Acute leukemias are a result of acquired mutations in early hematopoietic progenitors and are formed by the malignant transformation of hematopoietic stem cells. Therefore, the abnormalities can involve any hemopoietic lineages, including myeloid and lymphoid precursors, resulting in acute myelogenous or lymphoblastic leukemias. There is also a rare incidence of the involvement of a more committed stem cell with limited self-renewal capability. The changes include genetic abnormalities causing chimeric fusion genes, point mutations, or deletions [13,14,15].

Characteristically, acute leukemias are malignant, immature, poorly differentiated cells designated as blast cells. These have the potential for clonal expansion and proliferation, replacing the normal bone marrow cells. The lack of the normal development and function of the hematopoietic cells leads to the known characteristic clinical symptomatologies of leukemias. Acute leukemias are associated with the accumulation of undifferentiated blast cells in the marrow and other tissues. These replace and suppress the production of normal hemopoietic cells in the bone marrow. Early abnormal, non-functioning, undifferentiated myeloid or lymphoid progenitors accumulate in the bone marrow and peripheral blood and can infiltrate other tissues. Most reports indicate that the immune system plays a significant role in developing acute leukemias [16]. In children, early stimulation of the immune system has been suggested to reduce the risk of acute lymphoblastic leukemia [16,17,18,19,20,21,22,23]. Some reports hypothesize that conditions that cause early maturation of the immune system during infancy result in reduced chances of developing ALL later in life. A significant inverse relation between the occurrence of repeated early common infections, attending day care centers before age one, prolonged breastfeeding, and regular exposure to farm animals or pets and the development of ALL in the pediatric age group have been reported [24,25] Dysfunctional and decreased immunity in adults may contribute to compromised immunosurveillance and increased rates of cancer [26,27,28,29]. Immunosenescence, the gradual deterioration of the immune system brought on by aging, is proposed to increase the frequency and severity of malignancies. The progressive decline in immune function associated with aging results from cumulative alterations in B- and T-cell subpopulations, decreased circulating memory cells, T-cell dysfunction, and related changes [28,29].

### 2.1. Genetics

While not universally applicable, in some cases, there is evidence of a genetic predisposition to leukemia. Multiple genetic and environmental risk factors resulting in the development of leukemias are described, but there is no universally accepted, consistent cause for most cases. Therefore, the etiology of acute leukemias remains unknown. There are several genetic syndromes known to have higher susceptibility rates than usual for developing these disorders. Genetic diseases such as trisomy 21, Bloom syndrome, Klinefelter syndrome, ataxia telangiectasia, and disorders of telomers, as seen in Shwachman–Diamond syndrome, Fanconi anemia, dyskeratosis congenital, or germline mutations noted in CEBPA, RUNX1, and other background disorders, have higher rates of leukemia. Genetic rearrangements such as MLL/AF4 are reported to result in the development of acute leukemia [30,31,32]. Genetic and chromosome instability and exposure to certain trigger factors also have the potential to result in various leukemias [30,31,32]. Some genetic alterations may indirectly increase the occurrence of acute leukemia upon exposure to various environmental factors.

Several published reports suggest that, at least in a subset of acute leukemias in children, the disease originates during the perinatal period [33,34]. For example, in infants under one year of age with precursor B-cell ALL, rearrangement of the *NUTM1* gene (NUTM1r) is frequently seen [35]. A detailed investigation of this gene in the umbilical cord blood of infants may provide its pre-natal origins. It is proposed that in *ETV6-RUNX1* ALL, the recurrent secondary genetic events are mainly RAG-driven copy number deletions, and in the case of the high-hyperdiploidy form, these changes, with or without RTK-RAS mutation may occur [36]. It is reported that in the majority of ALL cases, there is involvement of the RTK-RAS pathway and histone modifiers. Single-cell tracking reports reveal that this mechanism is active throughout the leukemic evolution. The integration of data regarding point mutation and rearrangement discloses that ATF7IP and MGA tumor suppressor genes are involved in ALL [36,37,38,39,40,41,42,43]. Specific chromosomal translocations, such as those of *ETV6-RUNX1* (*TEL-AML1*), have been detected in the cord blood obtained at birth prior to the diagnosis of leukemia [34,44]. It is postulated that the ETV6-RUNX1 fusion gene, which is the molecular consequence of t (12;21) (p13;q22) and is present in approximately 25% of cases of childhood B-cell precursor ALL, is acquired in utero, but it requires additional post-natal somatic mutations in order to result in the development of overt leukemia. RTK-RAS fusion, which is also the molecular consequence of t (12;21) (p13;q22), is seen in approximately 25% of children with acute lymphoblastic leukemia (ALL). Studies have shown that this fusion alone is insufficient for initiating leukemia, and additional genetic changes for such development are required [42]. Therefore, RTK-RAS mutations, along with other changes, may be necessary for the development of high-hyperdiploid (51–67 chromosomes) ALL, which is a common pediatric B cell-precursor form of the disease. 

Over 90% of infants (<18 months) have a distinctive subtype of ALL with a pro-B immunophenotype and *MML-AF4* fusion genes. Whole-genome sequencing reveals few somatic changes, indicating that only a few mutations are needed to generate infant MLL-leukemia [45,46]. Somatic alterations of *IKZF1* are seen in the Philadelphia chromosome (Ph)-positive, PH-like, and DUX4-rearranged B-cell ALL [47,48]. In familial B-cell ALL, germline variants of *IKZF1* have been reported. In children with acute leukemia, deletion of IKZF1 has been observed [49,50,51,52,53,54,55]. According to genome-wide association (GWAS) studies, there are several non-coding variants associated with ALL. These variants are frequently at or near tumor suppressor genes or hemopoietic transcription factors. The risk of ALL development associated with these variants is relatively low. Germline genetic variation in ETV6 or PAX5 mutations can predispose an individual to ALL [56,57].

### 2.2. Leukemia in Twins

It is proposed that leukemia in twins is of pre-natal origin and initiates them to develop ALL or acute myeloblastic leukemia (AML) later in life [34]. Older twins aged 2–15 years often have more common subtypes of precursor B-cell ALL, and the concordance rate is significantly lower at approximately 15%, with pre-correction for the placental type. These age-associated differences, in concordance, prompted the suggestion that while infant ALL might be pre-natal in origin, the disease could be mostly post-natal in older children. Considering all evidence, it is postulated that in contrast with infant ALL, in older children, the disease may be post-natally initiated [37,58]. 

Twin birth constitutes approximately 1% of all deliveries, with half being monozygotic. A high concordance of ALL with specific infant immunophenotypic variants in identical twins has been reported [59]. This supports the pre-natal origins of *KMT2–AFF1* fusion in infantile ALL and suggests that leukemogenesis is complete by the time of birth. Other genetic abnormalities can also occur. It is postulated that infant ALL may be pre-natal in origin [37,45,46,59]. One piece of evidence for genetic predisposition to leukemia is concordant ALL in monozygotic twins. If one of the identical twins develops acute leukemia before the age of seven, the chance that the other would have the same disease is twice as much as that of the general population. Over time, this change gradually decreases, and by fifteen years of age, it becomes the same as the general population [60,61]. It is observed that, except in rare pairs with dichorionic but fused placentas allowing blood exchange, concordant ALL only occurs in monozygotic twins that are monochorionic. Thus, there is a single placenta [44]. The latter events occur in 60% of identical twins. Infants generally have pro-B immunotype and KMT2A fusion gene subtypes of ALL. In identical monochorionic, monozygotic twins diagnosed with ALL, whole-genome sequencing analysis indicated the genetic identity of initiating lesions and discordance for secondary genetic alterations, which may point to inter-twin variation in the uterine transmission of leukemia [62,63]. In identical twins concordant for ALL, specific secondary genetic alterations are found in each twin, which indicates separate post-natal evolutionary events [64,65]. No concordance has been found in dizygotic twins or for dissimilar cancers [66]. 

### 2.3. Carcinogenesis Attributed to Microbiome Flora

Several reports link microbiomes to cancer, including the effects of viruses, bacteria, and fungi [67,68,69]. Possible effects of viral infections such as Epstein Barr virus and human T-lymphotropic virus on the development of acute leukemias have been suggested. 

Fungal agents are found virtually in all environments, and many contain mycoviruses that are documented to be able to modulate various characteristics and genetics of their host [70,71,72,73,74]. This includes cessation of the production of aflatoxin by the host organism.

### 2.4. Mycovirus-Containing Aspergillus flavus and Acute Leukemia

The authors have shown that the products of mycovirus-containing *Aspergillus flavus* can alter the genetics of human cells, including those of normal and ALL and AML cell lines [75,76]. Furthermore, we have published that plasma from patients with acute lymphoblastic leukemia, unlike controls, has antibodies against this organism [77]. In addition, we have found that the exposure of mononuclear cells from patients with ALL in complete remission and long-term survivors to these products results in the redevelopment of genetic and cell surface markers characteristic of ALL [78]. In separate studies, we have documented that the exposure of normal and ALL cell lines to the products of the mycovirus-containing *Aspergillus flavus* results in the downregulation of several transcription factors known to be abnormal in this form of leukemia [75,76]. Other findings reveal that farmers and foresters who are most exposed to fungi have a higher rate of leukemia. In contrast, asthmatics have a lower rate, which may support our hypothesis that mycovirus-containing *Aspergillus flavus* has a role in the etiology of ALL. 

### 2.5. Fungi in the Environment

Fungi have a worldwide distribution and are known to cause human diseases, toxicities, and invasive pathogenicity, especially in immunosuppressed individuals. The carcinogenic effects of various fungal agents and their specific relation to the development of a variety of cancers in humans are well documented [79,80,81,82,83,84,85,86,87,88,89,90,91,92,93,94,95,96,97,98,99,100,101] (Table 1). Fungal organisms are also found to be a part of normal, non-invasive human flora. Fungi have a major significance in agriculture, representing certain health and commercial concerns. 

Fungi are ubiquitous microorganisms widely distributed in virtually every environment and have omnipresence in air, trees, plants, and animals. These organisms are found outdoors and indoors on surfaces, dust, compost heaps, dead vegetation, and air [102,103,104,105,106,107,108]. In nature, fungi have an important ecological role and are essential for degrading biological material in soil and elsewhere. In addition, they supply nutrients for and can be protective of trees and plants against other invading organisms. These organisms are found in soil, dust, compost heaps, and dead vegetation and can cause allergies [107,108]. The distribution of fungi widely varies based on the location, temperature, and environment. Generally, the amount of airborne fungal spores correlates with environmental factors such as increasing temperature, humidity, and the rate of precipitation [102,103,104,105,106]. Fungi can produce spores, which, depending on the environment, climate, and geographical location, can account for a significant portion of the air particulates. Fungal spores are found indoors and outdoors, with a generally larger presence in the latter environment [109,110,111,112,113,114,115,116,117,118,119,120,121,122,123]. In a study of 244 homes, the mean total indoor spore count was 4076/m [3], while outdoors, it was 8899/m [3] at the ground level [109]. Reports indicate that in the tropical rainforest air, which is usually rich in spores, these can account for 45% of the coarse particle mass (>1 μm) [123]. The density of spores is much less in urban areas and rural air, amounting to 4–11% of the fine particle mass (≤2.5 μm). The mean total spore count in mixed-evergreen forests has been reported to be twice as much as in coastal prairies. Spores’ density also depends on various factors, including season, temperature, air moisture, and other variables. For example, mean spore concentrations in the outdoor environment can be in the 50 spores/m [3] range in cold weather and increase to over 50,000 spores/m [3] of air in warm weather with increased moisture [107]. Thus, temperature and dew point are essential factors in the spores found in outdoor air [119,120,121,122]. It is estimated that regularly, there are 1000 and 10,000 fungal spores in every cubic meter of air. On average, an individual inhales 10,000 to 20,000 L of air per day, and generally, each breath contains 1–10 spores [123].

While all individuals are constantly exposed to fungi, some occupations are associated with increased exposure to mold [124,125,126,127,128,129,130,131,132]. For example, a farmer can inhale up to 750,000 spores per minute. While natural defenses such as sneezing or coughing can prevent dust or other particles from entering the lungs, due to their overwhelming numbers and small size, fungal spores can potentially bypass these defenses and barriers [124,125].

Fungal spores attach to airborne dust particles, and individuals working in farms or forests can inhale the combination [126]. Both in indoor and outdoor environments, *Aspergillus* is one of the most frequent fungi isolated from air samples [127,128,129,130,131,132].

A broad spectrum of effects for fungi, ranging from colonization with non-life-threatening mycobiome flora to severe lethal systemic infections, especially in immunocompromised individuals, has been reported. In addition, fungi have toxicity emanating from their products, which includes mutagenicity, carcinogenicity, growth impairment, and the stimulation of allergic effects. Generally, non-life-threatening infections by fungal agents in humans occur in the nails, skin, oral cavity, throat, and vagina [133,134,135,136]. Fungi constitute approximately 0.1% of the microbial DNA present in the gastrointestinal tract. Non-invasive, pan-cancer analysis has reported that some human samples harbor tumor-associated mycobiota in gastrointestinal and lung tumors. Various fungal species, including *Candida* and yeasts in the Dipodascaceae (*Galactomyces*, *Geotrichum*, and *Saprochaete*) family, are found colonizing the gastrointestinal tracts of healthy individuals. Filamentous and other fungal organisms are also found in various organs and systems of the human body. Some fungi, such as Penicillium and Debaryomyces species, enter the body through the diet or environment but, as a rule, do not colonize the organs. Severe and life-threatening fungal infections are often caused by *Aspergillus*, *Blastomyces*, *Candida*, *Coccidioides*, *Cryptococcus*, *Histoplasma*, *Mucoromycetes*, *Pneumocystis*, and *Talaromyces* species. The immune reaction to fungi is variable and ranges from absence of recognition to severe inflammatory responses with morbidity and mortality. Increased cutaneous fungal infections, especially with candida and Trichophyton rubrum and other infections with *Aspergillus* species, are found in older adults [137,138,139]. Associations of fungal infections with mortality in elderly individuals, who have lower immunity, in a variety of cancers, including esophageal, gastric, colorectal, lung, cervical, skin, and ovarian cancers, have been reported [138,139].

In an investigation of the fungi cultured from 295 samples of peripheral human lung and 2466 samples of sputum over a two-year period of time, 83% of the lung samples and 88% of the sputum samples had positive fungal cultures [140]. There was a significant number of *Candida albicans* cultured, amounting to 16% of the lung samples and 31% of the sputum samples [140]. A frequent occurrence of other fungi in the respiratory tract, including *Aspergillus fumigatus*, more frequent than it was distributed in the air, was detected [140].

While fungi can be a normal body flora, they can cause a number of mild to life-threatening infections or toxicities. In normal individuals, the immune response to fungi is highly variable, ranging from no reactions to severe responses. There is a wide range of diseases resulting from fungal infections, which, especially in immune-suppressed individuals can be severe, resulting in significant morbidity and mortality. The diseases produced by fungi range from mild nail, skin, oral cavity, throat, and vaginal infections to severe infections and sepsis, causing significant morbidity and mortality, especially in immune-suppressed patients. There is a broad spectrum of infections attributed to fungi ranging from intoxication and mild skin and oral lesions to severe life-threatening infections [141,142,143,144,145,146,147].

Fungi and their mycotoxins can be toxic to animals and humans, and their carcinogenesis is well documented [148,149,150]. Several mycobiomes have been implicated in the pathogenesis of various types of cancer. Murine and human investigations revealing the association of the fungi infiltrating the pancreas and producing pancreatic ductal adenocarcinoma (PDA) are available [151,152,153,154]. In several studies, *Malassezia* spp. has been linked to the development of PDA. Experimentally, the ablation of this mycobiome was found to be protective against tumor growth in PDA. In an experimental model, repopulation with this species accelerated oncogenesis. Unfortunately, in none of the studies were fungi associated with cancer tested for the existence of mycoviruses.

One of the mechanisms of tumor development due to fungi is proposed to be inflammation, including the activation of the C3 complement cascade. For example, based on a variety of experiments, the pathogenesis of pancreatic ductal adenocarcinoma is suggested to be due to the fungal migration of *Malassezia* from the intestinal tract to the pancreas. This process initiates a complement cascade through the activation of mannose-binding lectin (MBL) [146,155]. The extracellular MBL then recognizes an unidentified carbohydrate, which is expressed by *Malassezia* and activates the C3 protein. This results in an inflammatory immune response termed the complement cascade. Reports indicate that ligation of MBL, which binds to glycans of the fungal wall to activate the complement cascade, is necessary for the progression of oncogenesis. On the contrary, oncogenic deletion of MBL or C3 in the extra-tumoral compartment, or the knockdown of C3aR in tumor cells, can potentially be protective against the development of the tumor [155].

Fungi can be involved in human and animal tumorigenesis through multiple other pathways. These involve a number of variables, including, but not limited to, the type of fungal organism, toxin production, bioactive factors, microbiome interactions, host factors such as immunity, genetic and epigenetic background, bioactive factors, microbiome interactions, and others [133,134,135,136]. As noted before, certain types of cancer, including skin, lung, esophageal, gastric, colorectal, cervical, ovarian, prostate, and other cancers, have been reported to be associated with a variety of fungal organisms [82,95,156,157,158,159,160,161,162,163,164,165,166,167,168,169,170]. 

Reports detecting higher rates of fungal DNA in cancer tissues are available [156,157]. While there is an increased rate of lung cancer in patients with asthma, as described below, having allergies is also reported to be associated with a decreased risk of several other malignant disorders. Primary fungal agents found to be associated with carcinogenesis are *Candida* (*albicans*, *glabrata*, *tropicalis*, *krusei*, *parapsilosis*, and *neoformans*), *Fusarium* (*verticillioides* and *proliferatum*), and *Aspergillus* (*flavus* and *parasiticus*). The diseases formed include lung, esophageal, gastric, pancreatic, colorectal, cervical, ovarian, and skin cancers (Table 1). An example of the carcinogenic potential of fungi is that of *Candida albicans.* This organism can promote cancer through several mechanisms, including triggering inflammation, producing carcinogenic byproducts, initiating the T-helper type 17 (Th17) cell response, and others [158,159]. It should be noted that *Candida* species are reported to be associated with gastrointestinal cancers [156].

Several mechanisms for the effects of fungi on the initiation of cancers have been suggested but not fully explored. No data regarding mycovirus-infected fungi and carcinogenesis, except for leukemia, in any age group are currently available [75,77,78,171,172]. In our view, some evidence supporting the involvement of fungal agents in leukemogenesis is the reduced rate of certain acute leukemias in asthmatics and their increased rate in farmers and foresters, as described below.

### 2.6. Incidence of Cancer and Leukemia in Asthmatics

Asthma can be associated with fungi and triggered by fungal spores in the environment. Severe asthma attacks have been associated with a sudden increase in the concentration of spores in the air [173,174]. Based on a review of the literature, there is a relationship between the degree of fungal sensitivity and the severity of asthma [173,174,175]. Exposure to dampness, which facilitates fungal growth, is shown to cause an increased rate of asthma [176]. Epidemiological studies reveal that many asthmatics are allergic to fungal species, including *Alternaria*, *Aspergillus*, *Cladosporium*, and *Penicillium* [177]. The therapeutic value of antifungal agents in patients with severe asthma and fungal sensitivity has been examined [178]. The relationship between allergies, atopies, asthma, and the development of cancer, including leukemia, has been extensively explored [179,180,181,182,183,184,185,186,187,188,189,190,191,192,193,194,195,196,197,198,199,200,201,202,203,204,205,206,207,208,209]. It is of interest that some, but by all means not all, [179,181] studies suggest that asthmatic patients may have a lower incidence of some malignant disorders [181,182,183], especially acute leukemias [184,185,186,187,188]. While in asthmatic patients, the rate of some malignant disorders, especially lung cancer, is increased, in several studies, asthma and allergies are found to be inversely associated with the risk for non-Hodgkin’s lymphoma and acute lymphoblastic (ALL) and myelogenous (AML) leukemias [182,183,190,191,192,193]. In 77,952 asthmatic patients, there was an increase in the occurrence of several solid tumors. However, the rate of lymphatic leukemia was found to be reduced [183,190]. 

It appears that, compared with the general population, individuals with allergies generally have a decreased risk for some forms of malignant solid tumors [196,197,198]. One review found that allergic patients have a noticeably reduced risk of glioma, colorectal cancer, cancer of the larynx, non-Hodgkin lymphoma, and oral, esophageal, pancreatic, gastric, and uterine body cancers [196]. The same study, however, found no associations or conflicting data regarding breast, leukemia, lung, melanoma, and thyroid cancers [196]. Overall, although, due to a lack of consistency, it is not possible to draw a uniform conclusion on the relationship between allergies and cancer of all sites, there appears to be an inverse association between acute lymphoblastic (ALL) and myelogenous leukemia (AML) [182,183,186,187,188,191,192,193,199,200,201,202,203,204,205], non-Hodgkin’s lymphoma, pancreatic cancer, and glioma. In contrast, lung cancer is positively associated with asthma. Additional studies are needed to better understand the relationship between other neoplasms and allergic conditions [182,183,197]. 

In most studies, asthmatic patients, mainly if diagnosed for at least 10 years, have reduced risks of hematological malignancies [183,197,198,205,208]. The odd ratios associated with asthma were below unity for most hematological malignancies. After a period of 10 years post-diagnosis for asthma, the odd ratios for the acute leukemias were reported to be 0.6 (95% CI: 0.4–0.9; 26 exposed cases) and 0.4 for the acute myeloid leukemia (95% CI: 0.2–0.8; 9 exposed cases). A significantly lower risk for ALL (OR = 0.6; 95% CI: 0.3–1.0; based on 10 exposed cases) was reported [199]. In a study of 1,102,247 patients with asthma and/or hay fever who were cancer-free at the baseline, during 18 years of follow-up, 81,114 cancer deaths occurred [197]. A significant inverse association between the history of asthma or hay fever and overall cancer mortality was found. A history of asthma is reported to be associated with a significantly lowered risk of mortality from leukemia [206,207]. The negative trend of association of atopy with leukemia appears to be more apparent in children [186,187,188,193,199,200,201,202,206,207]. Also, a consistent inverse association between asthma and the development of acute lymphoblastic leukemia has been reported in the pediatric age group. Considering the fact that many asthmatics are allergic to fungi, this may indicate that the existence of antibodies and reactions by the immune system can result in the detection and elimination of the elements that may be responsible for alterations that may be responsible for some forms of cancer.

The association of asthma, particularly after 10 years of diagnosis, with a reduced risk of some hematological malignancies, such as ALL, supports the immune surveillance hypothesis [210,211]. Such a hypothesis aligns with our prior reported research [75,76,77] and current hypothesis.

Several investigations indicate that a combination of genetic predisposition, infections, and exposure to various environmental hazards influences the incidence of acute leukemias. While the effects of genetic and environmental factors have been suggested [209], so far, no consistent infectious etiology has been identified. For the latter, the role of fungal agents, with and without mycoviruses, needs to be further investigated.

### 2.7. History of Allergy and Mortality from Cancer

Several studies suggest a limited inverse association between a history of allergy and mortality from cancer [206,207]. In the evaluation of patients with hay fever and asthma, modestly lower mortality from colorectal cancer has been detected.

### 2.8. Agricultural Workers and Foresters and Leukemia

While various factors may be involved, and reports are inconsistent, several studies indicate that workers in agriculture and forestry may be at increased risk of various malignant disorders, including leukemias [210,211,212,213,214,215,216,217,218,219,220,221,222,223]. The factors responsible for the increased risk of cancers, including hematologic malignancies, in farmers and foresters are not entirely clear. Exposure to pesticides, chemicals, or viruses and prolonged antigenic stimulus leading to lymphoproliferation has been suggested. It is of note that farmers and foresters are exposed to the soil, farm, and forest air, which is known to contain much greater concentrations of fungi and spores [224,225,226,227,228,229,230], some of which contain mycoviruses. Currently, most studies arbitrarily relate the increased rate of cancer and leukemia in farm workers to exposure to chemicals [210,211,212,213,214,215,216,217,218,219,220,221,222,223]. No research regarding exposure to mycovirus-containing fungi in this group is available. While not confirmed in all studies, some reports indicate a higher risk of childhood leukemia in children born in agricultural areas [231,232,233,234,235,236]. This is also often blamed on exposure to pesticides [237]. The effects of other environmental factors, including the high density of fungi and spores in farm areas, have not been evaluated [238].

## 3. Conclusions and Hypothesis

**Figure 1:**
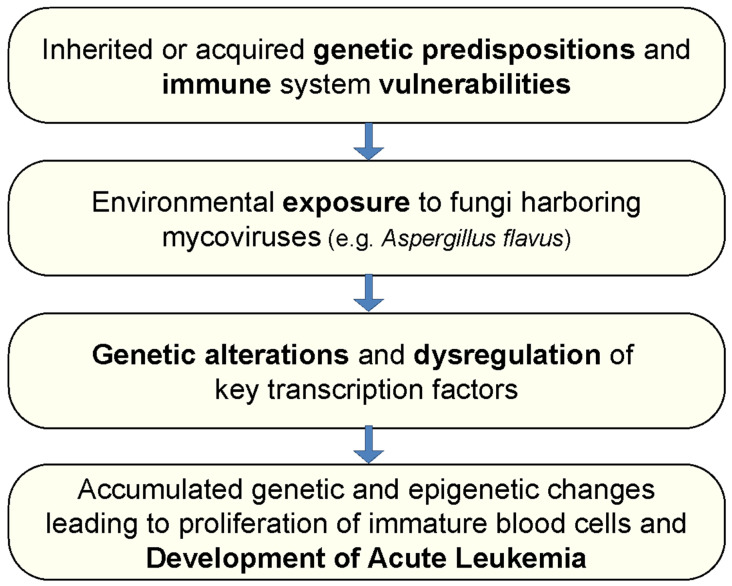
Hypothesis for the sequence of development of acute leukemias under the influence of mycovirus-containing *Aspergillus flavus*.

The studies outlined in this manuscript may point to certain fungi’s ability to induce carcinogenesis, especially leukemogenesis. This is in line with our prior published reports, which point to a certain mycovirus-containing aspergillus flavus as a cause of acute leukemias. As noted before, our investigations revealed that patients with ALL uniformly have antibodies to the products of a certain mycovirus-containing Aspergillus flavus (MCAF), which we isolated from the home of a patient with this disease [77]. Furthermore, exposure of mononuclear blood cells from patients in complete remission, or long-term survivors, to the products of this organism resulted in the redevelopment of the genetic changes and cell surface phenotypes characteristic of ALL [78]. In addition, we reported that the incubation of normal and ALL cell lines with the products of MCAF resulted in genetic changes and alteration in transcription factors, known to be of significance in this disease [75]. It is of interest that mycoviruses are known to alter the genetics of their fungal hosts [73,74]. We hypothesize that it is likely that mycoviruses in fungal agents have similar effects on human cells, changing the cellular genetics of genetically vulnerable individuals and resulting in leukemogenesis and carcinogenesis. The summary of our hypothesis is shown in Figure 1. The fact that acute leukemias are most frequent in individuals who work in environments that contain large amounts of fungi, such as foresters and farm workers, and is less than average in asthmatics, who generally have antibodies to fungi, is in line with the above hypothesis.

## Figures and Tables

**Table 1:** Fungal infections associated with various carcinogenesis (**species** and strains) [79,80,81,82,83,84,85,86,87,88,89,90,91,92,93,94,95,96,97,98,99,100,101].

Cancer Type	Fungi Associated with Cancer
**Skin cancer**	***Candida* species:** *C. albicans*, *C. cladosporioides*, *C. glabrata*, *C. parapsilosis*, *C. tropicalis*; **Alternaria species:** *A. alternata*, *A. infectoria*, *M. arundinis*, *E. oligosperma*
**Lung cancer**	***A. fumigatus***, ***Cryptococcus* sp.**, ***Fusarium***, ***H. immitis***, ***Histoplasma capsulatum***, ***P. jiroveci***, ***Pneumocystis* sp.**, ***Rhizopus***, *Talaromyces marneffei*, ***Trichosporon***
**Oral cancer**	***C. albicans***
**Esophageal carcinoma**	***Aspergillus* sp.:** *A. flavus*, *A. parasiticus*; **Candida sp.:** *C. albicans*, *C. glabrata*, *C. tropicalis*, *C. krusei*, *C. parapsilosis*; ***Fusarium* species**.: *F. verticillioides*, *F. proliferatum*; **Torulosis sp.:** *T. glabrata*, *T. tomata*
**Gastric cancer**	***Aspergillus* spp.**, ***Blastomyces* spp.**, **Candida sp.:** *C. albicans*, ***Coccidioides* spp.**, ***Cryptococcus* spp.**, ***Fusarium* spp.**, ***Histoplasma* spp.**, ***Malassezia* spp.**, ***Mucor* spp.**, ***Paracoccidioides* spp.**, ***Penicillium* spp.**, ***Phialemonium* spp.**, ***Rhodotorula* spp.**, **Saccharomyces *cerevisiae***, ***Trichosporon* spp.**
**Colorectal cancer**	***Aspergillus* sp.:** *A. flavus*, *A. sydowii*, *A. ochraceoroseus*; **Candida sp.:** *C. albicans*, *C. tropicalis*, ***Cladosporium***, ***Cryptococcus***, ***Debaryomyces fabryi***, ***Histoplasma***, ***Kwoniella mangrovensis***, ***Malassezia globosa***, ***Moniliophthora perniciosa***, ***Paracoccidioides***, ***Phoma***, ***Pneumocystis***, ***Plectosphaerella***, ***Pseudogymnoascus* sp.**, ***Rhodotorula***, ***Scedosporiosi***, ***Talaromyces islandicus***, ***Trichosporon***, ***Thanatephorus***, ***Zygomycetes***
**Cholangiocarcinoma**	***Aspergillus* sp.:** *A. flavus*, *A. parasiticus*, *Penicillium*; **Candida sp.:** *C. albicans*, *C. glabrata*, *C. tropicalis*, ***Penicillium***
**Pancreatic ductal adenocarcinoma**	*Malassezia*
**Breast cancer**	*Aspergillus*, *Candida*, *Coccidioides*, *Cunninghamella*, *Geotrichum*, *Pleistophora*, *Rhodotorula*, *Filobasidiella*, *Mucor*, *Trichophyton*, *Epidermophyton*, *Fonsecaea*, *Pseudallescheria*, *Penicillium*, *Ajellomyces*, *Alternaria*, *Rhizomucor*, *Piedraia*, *Malassezia*
**Cervical cancer**	*Candida*, *Cryptococcus laurentii*, *Gjaerumia*, *Pleosporales*, *Malassezia*, *Nakaseomyces*, *Sporidiobolacea*, *Saccharomyces*
**Ovarian cancer**	*Pneumocystis*, *Acremonium*, *Cladophialophora*, *Malassezia*, *Microsporidia Pleistophora*, ***Ajellomyces***, ***Aspergillus* sp.**, ***Candida* sp.**, ***Cladosporium***, ***Coccidioides***, ***Cryptococcus***, ***Cunninghamella***, ***Issatchenkia***, ***Nosema***, ***Paracoccidioides***, ***Penicillium***, ***Pleistophora***, ***Rhizomucor***, ***Rhizopus***, ***Rhodotorula***, ***Trichophyton***
**Prostate cancer**	*Aspergillus* sp., ***Candida* sp.:** *C. neoformans*, *C. immitis*, *H. capsulatum*, *B. dermatitidis*

## Data Availability

The literature supporting this study’s findings is available online or in print.
